# We Move or Are We Moved? Unpicking the Origins of Voluntary Movements to Better Understand Semivoluntary Movements

**DOI:** 10.3389/fneur.2022.834217

**Published:** 2022-02-21

**Authors:** Sasivimol Virameteekul, Roongroj Bhidayasiri

**Affiliations:** ^1^Department of Medicine, Faculty of Medicine, Chulalongkorn Centre of Excellence for Parkinson's Disease & Related Disorders, Chulalongkorn University and King Chulalongkorn Memorial Hospital, Thai Red Cross Society, Bangkok, Thailand; ^2^The Academy of Science, The Royal Society of Thailand, Bangkok, Thailand

**Keywords:** semivoluntary movement, tics, functional movement disorders, stereotypy, perseveration, compulsion, utilization behavior, mannerism

## Abstract

The capacity for voluntary control is seen as essential to human movements; the sense that one intended to move (willing) and those actions were self-generated (self-agency) gives the sense of voluntariness and of being in control. While the mechanisms underlying voluntary movement have long been unclear, recent neuroscientific tools have identified networks of different brain areas, namely, the prefrontal cortex, supplementary motor area, and parietal cortex, that underlie voluntary action. Dysfunction in these brain areas can result in different forms of semivoluntary movement as the borderland of voluntary and involuntary movement where a person may experience a disordered sense of will or agency, and thus the movement is experienced as unexpected and involuntary, for an otherwise voluntary-appearing movement. Tics, functional movement disorders, stereotypies, perseveration, compulsions, utilization behaviors, and motor mannerism have been described elsewhere in the context of psychoses, and are often mistaken for each other. Yet, they reflect an impairment of prefrontal cortices and related circuits rather than simple motor systems, which results in the absence of subjective recognition of the movements, in contrast to other neurological movement disorders where principal abnormalities are located within the basal ganglia and its connections. Therefore, their recognition is clinically important since they are usually associated with neurodevelopmental and neurodegenerative disorders. In this review, we first defined a conceptual framework, from both a neuroanatomical and a neurophysiological point of view, for the generation of voluntary movement. We then examined the evidence linking dysfunctions in different motor pathways to each type of movement disorder. We looked at common semivoluntary movement disorders providing an overview, where possible, of their phenomenology and brain network abnormalities for each condition. We also emphasized important clinical feature similarities and differences to increase recognition of each condition in practice.

## Introduction

Human movements can be broadly divided into two types: those associated with intentional action (intentional or voluntary movement) and those without intention, which includes normal non-intentional movements, reflex (response to external signal) and involuntary movement (1). Voluntary movements are self-generated, willed actions performed as a result of cognitive processes (2). While non-intentional movements, on the other hand, refer to body movements outside of one's intention (3). It includes several kinds of movements. Non-intentional normal movements in which the movements are not done by one's intention, but often naturally occur without causing problems in daily life (e.g., associate movements, mirror movements, yawning, etc.) (4). Reflexes are another normal, non-intentional activity in response to stimuli (5). Several reflexes that affect movement can be classified as proprioceptive reflexes, which originate from receptors within muscles, tendons, and joints (e.g., stretch reflex and tendon reflex); and exteroceptive reflexes, which originate from afferent input from the skin and subcutaneous tissue (e.g., extensor-thrust reflex, flexor reflex, and crossed extensor reflex) (6). The last one is pathologic involuntary movements, in contrast, abnormal movements which are considered to be treated as a symptom of disorders (e.g., tremor, dystonia, chorea, etc.) (3). This subcategory also includes involuntary movements that appear during voluntary activity (e.g., mirror movements and synkinesias) (7). It can be noted that mirror movements have been considered either physiological, presenting in healthy children and can be elicited in adults under conditions of intense physical activity, movements involving large force generation, and proximal muscle use (8, 9); or pathological when mirror movements persist in the adult with age-related neurological diseases (e.g., stroke, amyotrophic lateral sclerosis, Parkinson's disease, etc.) (10–12).

**Figure 1:**
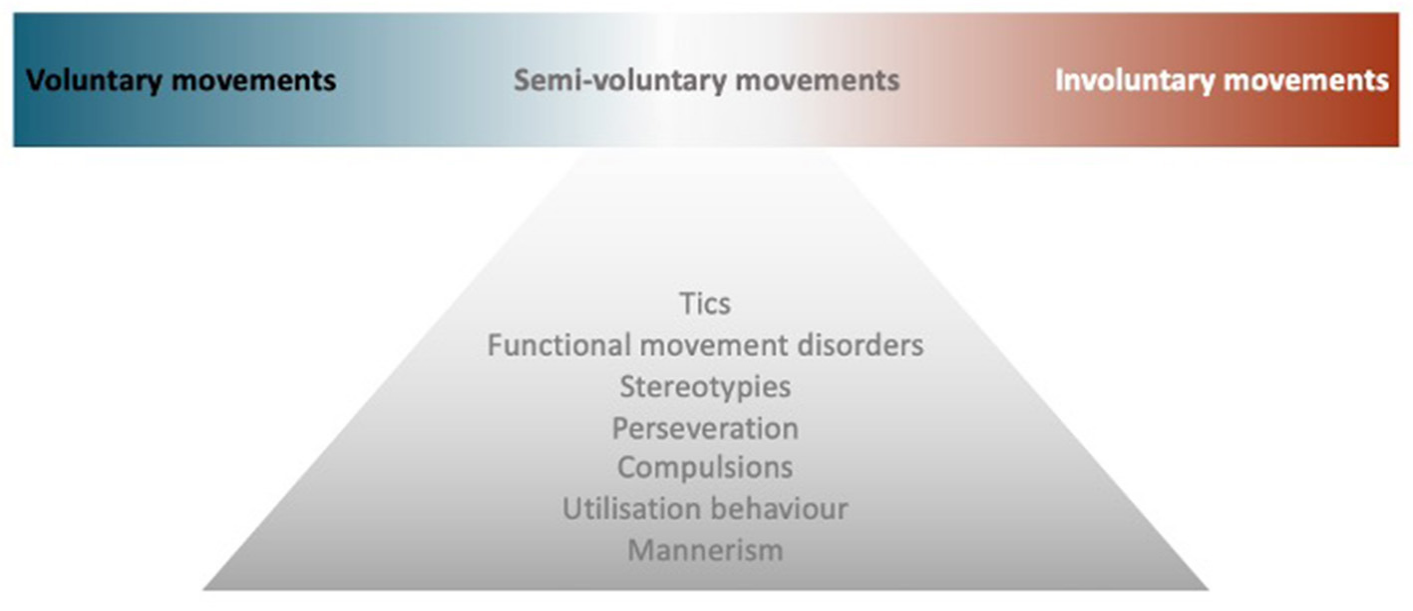
Model of semivoluntary movement. A diagram illustrates the concept of semivoluntary movement which is the neurological conditions in the borderland between voluntary and involuntary movements.

Though the voluntary movement is a fundamental component of human functioning, what exactly happens in the brain when we decide to move remains unclear. This review will first highlight key mechanisms in the generation of voluntary movement, with insights into the nature of volition (How to define voluntary action), then explore neurological conditions in the borderland between voluntary and involuntary movements, i.e., semivoluntary movement (Figure 1). Finally, we look at some behaviors that, by clinical features, can mimic repetitive semivoluntary movement.

## How Do We Move?

**Figure 2:**
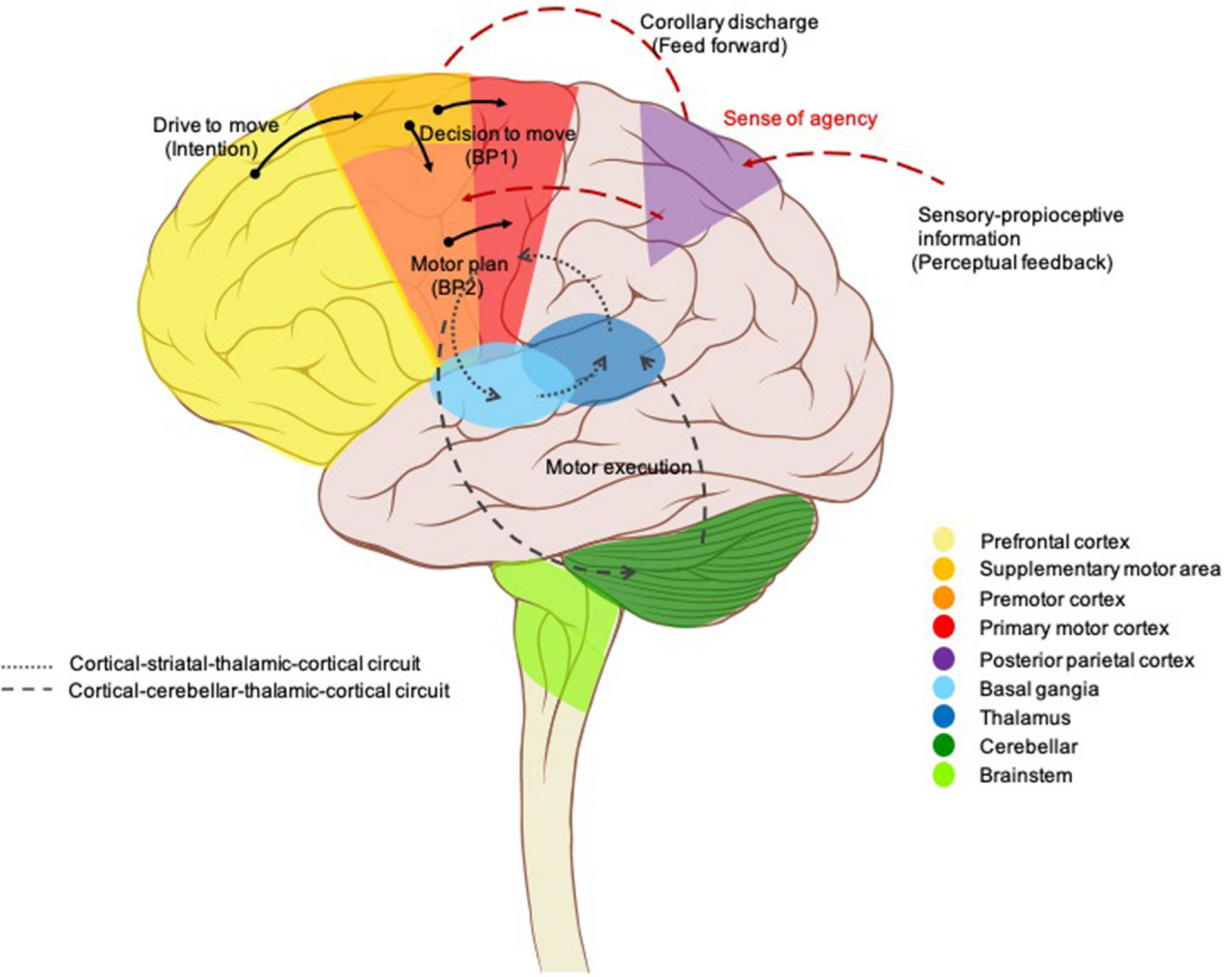
Brain circuits control voluntary movement. Voluntary movements are executed consciously under the control of the brain, starting with the intention to move created in the prefrontal cortex and limbic area. Next, presupplementary and supplementary motor areas are involved in programming the complex sequences of movements required. The premotor cortex primarily selects movements based on external information from the parietal cortex, it also contributes to some aspects of voluntary movement. The presupplementary and supplementary motor areas; together with the premotor area generate the readiness potentials (Bereitschaftspotential 1). This information is sent down *via* the motor cortex to two side loops—basal ganglia and cerebellum—for checking and modulating motor control. Then, it needs to be conveyed to the motor cortex again passing through the thalamus. A corollary discharge is also created in parallel (feedforward model) and then sent to the parietal cortex for comparison with the proprioceptive feedback, resulting in a sense of agency. Finally, the neural signal leaves the primary motor cortex (Bereitschaftspotential 2) for the spinal cord and contralateral muscles to trigger the actual movement.

Neuroanatomical conceptions of motor pathways, initially described by (13), centered on “final common pathways” through the primary motor cortex to the lower motor neurons of the spinal cord which have long axons traveling along peripheral nerves to innervate skeletal muscles (13, 14). All the movements reflect the interaction of these supraspinal commands along with sensory inputs and spinal cord interneurons. Moreover, these final common pathways are greatly regulated or modulated by two feedback loops; the basal ganglia loop and the cerebellar loop, which play an important role in a variety of movements. Voluntary movement is, by definition, the intended execution of an action that is the result of cognitive processes (1). In other words, voluntary movement is a product of the coordinated operation of various neural systems and is essential for flexibly achieving a particular goal. The prefrontal cortex (PFC) and limbic area, thus, have been demonstrated to be important structures for executive functions (15, 16) in which they initiate an intention to move and determines what kind of movement is required (goal-directed action; drive to move), then the presupplementary or supplementary motor area (SMA) makes an actual plan for the movement including order, sequence, and timings (internal preparation for movement; the decision to move) (17, 18). On the other hand, the premotor cortex primarily selects movement based on external information from primary sensory cortices, with is, in turn, transferred to the parietal cortex and premotor cortex, respectively. The parietopremotor-primary motor (M1) circuit is involved when motor actions depend on external cues, i.e., when actions are driven by sensory stimuli (19). Pre-SMA-M1 and parietopremotor-M1 circuits are normally balanced allowing healthy humans to effortlessly perform daily movements, but without unintentionally touching or reacting to every object from their surroundings (7). This information is then sent down *via* the motor cortex to two side loops—basal ganglia and cerebellum—for checking and modulating motor control. Then, it needs to be conveyed to the motor cortex again passing through the thalamus. Finally, M1 sends the final messages for the actual movements (motor execution) to some subcortical structures, such as the reticular nucleus, vestibular nucleus, red nucleus, spinal motoneurons, and so on (20) (Figure 2).

Neurophysiological studies, to date, have generally focused on a theoretical framework of voluntary movement as a multilevel process that incorporates internal prediction (feedforward) with external cues (feedback) (21). Figure 2 illustrates this proposed model. A cortical process starts with a drive to move, as a result of limbic, homeostasis, and goal-directed processes. The goals or outcomes of the voluntary action are more varied and more complex in humans than in animals. In animal studies, goal-directed actions typically involve the search for biologically important goals (e.g., food, water, etc.). While voluntary actions in humans may be directed at more abstract goals, or higher needs (22). The SMA is involved in the process of planning for purposeful actions (e.g., throwing a ball, rising from a chair) (18, 23). The network of cortical areas responsible for the motor preparation process was further investigated by Cunnington et al. (24) who demonstrated that the SMA, pre-SMA, anterior cingulate cortex (ACC), and premotor cortex actively generate the early component of Bereitschaftspotential (BP) or readiness potential BP1 which linked to motivational, intentional, and timing properties (25–27). In addition, the late component (BP2) of the BP, arising from the M1, was found to be linked to motor execution and performance (27–29). Whenever a voluntary movement is made, an efferent signal is produced and sent to the motor system, a copy of the predicted sensory signal, known as an efference copy (corollary discharge), is also created in parallel, by providing the input to a feedforward model, and then sent to the brain regions that receive perceptual input for comparison with the proprioceptive feedback after completion of the actions (21, 30). This process has been localized to the temporoparietal junction (TPJ), PFC, and the cerebellum (31–33). The purpose is not only to rectify movement accuracy but also is the basis of our sense of agency (SoA), the feeling of control over one's own actions and their consequences in the external world (34) which is a fundamental aspect of voluntary movements, as it allows us to distinguish between those sensory consequences that we cause, and those are external generate (35). SoA is generated when the predicted effect of the action (feedforward) matches the actual effect (feedback) (36, 37). In contrast, if a mismatch is detected, the movement is generated without an associated SoA, and thus can be perceived as externally generated (38, 39), a phenomenon known as loss of sensory attenuation (40, 41), which may explain why patients report that they do not experience the self-pace movement as voluntary (42).

## Disorders of the Motor System

Based on the above mechanisms for voluntary movement, any damage of the final common pathways usually causes paralysis or paresis (20). Dysfunction can be in the upper motor neurons, for example, primary motor cortex, subcortical structures, brainstem, and corticospinal tracts; or lower motor neurons, which are alpha motor neurons of the spinal cord, peripheral nerves, muscles, or neuromuscular junctions (43). Movement disorders caused by defects in the basal ganglia loop are called extrapyramidal disorders with specific localizations within the basal ganglia, classically associated with certain movement disorders: substantia nigra with bradykinesia and rest tremor; subthalamic nucleus with ballism; caudate nucleus with chorea; and putamen with dystonia. Pathology of the cerebellum loop, or its pathways, is typically characterized by impairment of coordination (asynergy and ataxia), misjudgment of distance (dysmetria), and intention tremor (3, 44).

**Figure 3:**
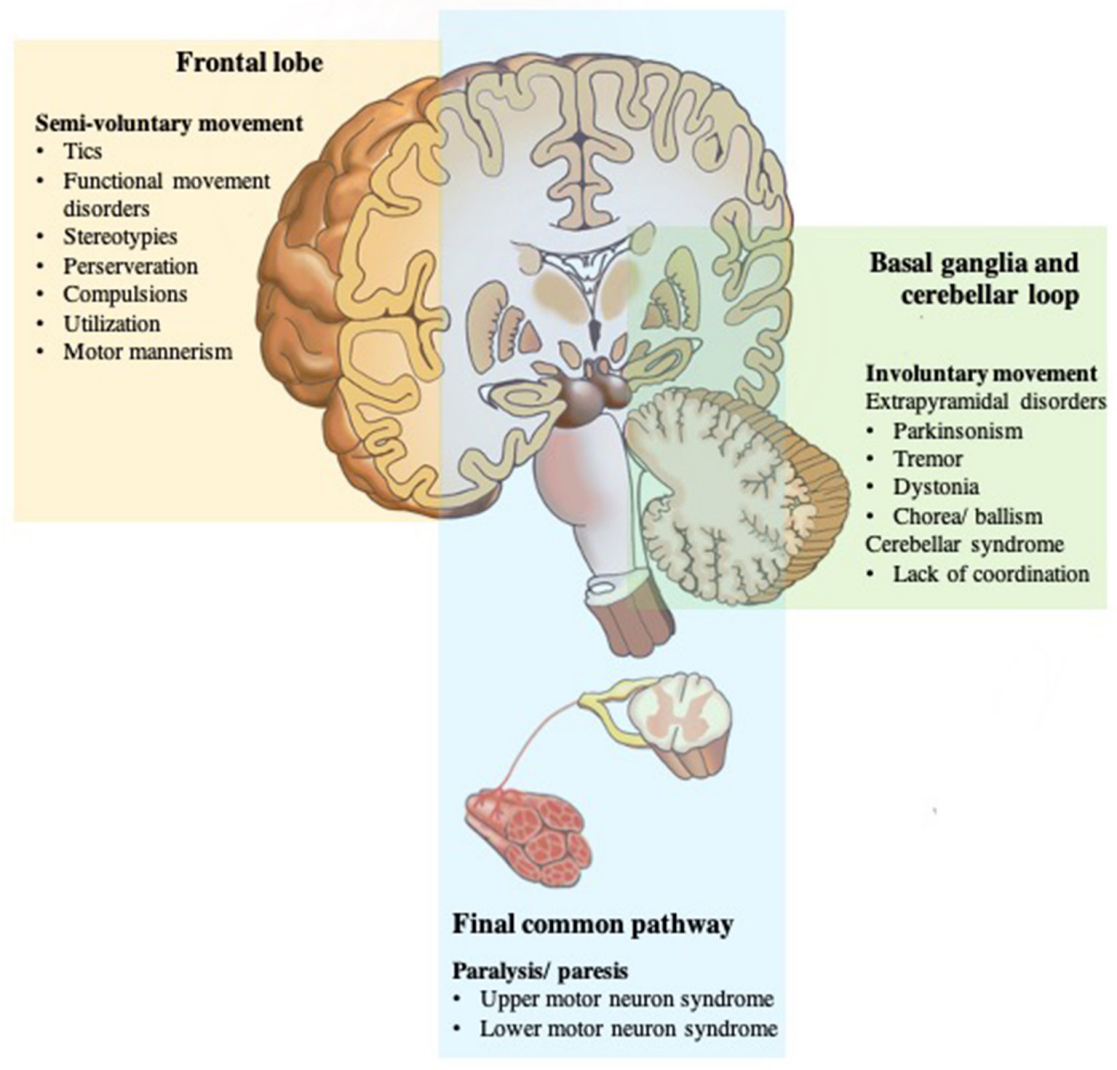
Disorders of the motor system. The motor system is deconstructed into component parts, but they work together to produce movement that we take for granted until there is a problem. Any damage of the final common pathways (shown in blue) can cause upper or lower motor neuron syndromes resulting in an inability to move due to paresis or paralysis. Dysfunction of the two parallel loops (shown in green) disturbs smooth movements resulting in pathologic involuntary movement disorders; basal ganglia loop dysfunction with extrapyramidal disorders; cerebellar loop dysfunction with a lack of coordination. While any damage of the frontal lobe or final common pathways input (shown in yellow) appears to involve self-initiation and conscious awareness of movement, and thus, results in no sense of voluntariness for an otherwise voluntary-appearing movement.

However, there are some exceptions to this general rule, for example, myoclonus does not appear to be mainly related to basal ganglia pathology, and often arise elsewhere in the central nervous system, namely, the cerebral cortex (cortical reflex myoclonus), brainstem (reticular reflex myoclonus, hyperekplexia, and rhythmical brainstem myoclonus such as palatal myoclonus and ocular myoclonus), and spinal cord (segmental myoclonus and propriospinal myoclonus) (3). Finally, any damage at the final common pathways input (higher motor disorders), namely, the PFC and related regions, namely, premotor cortex, SMA, pre-SMA, ACC, and parietal areas, appear to involve self-initiation and conscious awareness of movement (7). When individuals perform the movement without experiencing the feeling of ownership, then there is no sense of voluntariness concerning the movement, thus they feel quite similar to physically involuntary movements (45, 46) (Figure 3).

**Figure 4:**
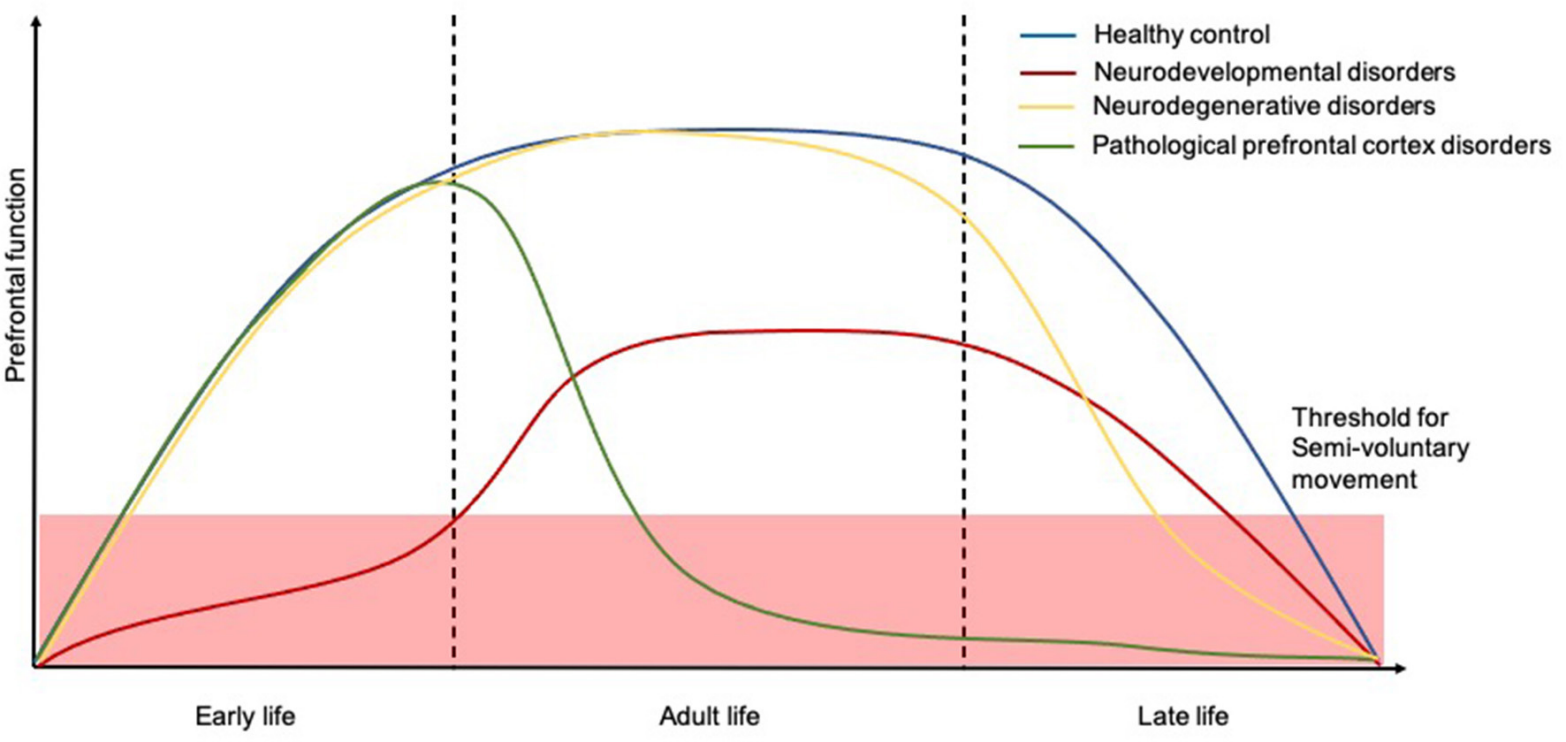
Temporal trajectories of the relationship between human prefrontal function and course of semivoluntary movement. The *x*-axis shows time and the *y*-axis shows the relative human prefrontal function. The schematic illustrates how prefrontal function in people with neurodevelopmental disorders (red), neurodegenerative disorders (yellow), and other pathological prefrontal cortex disorders (green) might decline, for which the semivoluntary movement (under the threshold) will begin to emerge compared to healthy population (blue).

Capacity for voluntary action is gradually developed across childhood to reach a plateau where a well-functioning motor system permits well-controlled fluid movement. However, certain repetitive semivoluntary movements can be part of normal motor development suggesting an etiological basis of incomplete cortical control of endogenous patterning in maturing neuromuscular pathways (47). These movements appear to go through a normal progression in normal developing children, eventually disappearing and being replaced by more complex voluntary movements concerning daily activities and ordering of objects as a result of full frontal cortex development and maturation (48). On the flip side, increasing age accompanied by a progressive decline in the frontal cortex function, and certain neurological and developmental disorders, can cause these pathological semivoluntary movements to re-emerge (Figure 4). In the following section, we will cover common semivoluntary movement disorders with an overview, where possible, of the phenomenology and anatomy and neuropathophysiology of each condition. We also emphasize important clinical features similarities and differences to increase recognition of each condition in practice.

## Disorders of Semivoluntary Movements

### Tics and Tourette Syndrome

Tics are hyperkinetic movement disorders characterized by sudden, brief, intermittent, and repetitive movements or vocalizations which appear seemingly uncontrollable, out of context, and exaggerated of normal movement (49, 50). Tics have intra- and interpersonal phenomenological variability which can be simple or complex movements, or even stereotyped, but look like movements that can be made voluntarily or can be mimicked easily by the patients. Simple tics are defined as movements involving a single muscle or muscle group and appear as a purposeless jerk (e.g., eye blinking, nose wrinkling, head jerking, and shoulder shrugging) or sound (e.g., grunting, throat clearing). Complex tics, on the other hand, are more coordinated and often resemble goal-directed movements (or sounds), but lack an obvious purpose and appear repetitively with an inappropriate intensity and frequency (51). Further typical features in complex tics were pali-, echo-, and coprophenomena. Paliphenomena indicates the repetition of words or phrases (palilalia) or motor acts (palipraxia) (52). Echophenomena indicates the imitation of movements (echopraxia) and vocalizations (echolalia) (53), while coprophenomena represent obscene, offensive, or other socially inappropriate behaviors (verbal—coprolalia and gestures—copropraxia) (54). When multiple motor and vocal tics are present in a patient, beginning before the age of 18 years and present for more than 12 months in the absence of secondary causes, the criteria are met for a diagnosis of Tourette syndrome (TS) (55). The majority of patients with TS present with comorbidities of attention deficit hyperactivity disorder (ADHD), obsessive-compulsive disorder (OCD), anxiety disorders, and depression (56).

Tics resemble voluntary actions in that they share many of their neurophysiological characteristics. First, conscious awareness of tics is facilitated in most cases by a premonitory urge, a sensory phenomenon (urge, itch, tingling, tension, feeling, or other sensation) that appears before the occurrence of the tic. The sensation often intensifies until the movement is performed, and is relieved following the completion of the movement (50, 57). Second, tics can be completely or partially inhibited on demand. Moreover, patients may report varying levels of voluntariness, they are often unsure whether the movements just happening or done intentionally. Tics are thus located in the borderland between voluntary and involuntary actions.

Neuroscientific tools to explore brain structure, excitability, and connectivity have come a long way over the last decade which has allowed new insights into brain function in patients with tics. Neuroimaging, including structural MRI, metabolic PET, and functional MRI (fMRI), demonstrated structural and functional abnormalities at different levels over and above the hyperactive corticostriatothalamocortical (CSTC) loops, a series of circuits that are involved in the generation of voluntary action, which were previously believed to be involved in the generation of tics. Recent studies have readjusted the pathophysiology from the subcortical, striatothalamic, to the primary sensorimotor cortical level including premotor, primary motor, and sensory areas (58–61). It is suggested that more recent generalized neuroanatomical dysfunction may account for heterogeneity across clinical manifestations, psychopathology, and associated behaviors (Figure 2) (56, 58).

Electrophysiological studies have been performed in patients with tics to determine brain activity. In the late 20th century, it was reported by Obeso et al. that there was no premovement electroencephalogram (EEG) potential or motor-related cortical potential (MRCP) before simple tics, while they were present in voluntary imitated tics (62). These findings suggested that tics differ from voluntary movements due to a lack of cortical preparatory activity. More recent work, on the other hand, demonstrated MRCPs before tics and the imitations, but early BP components (BP1) were not seen (63). Interestingly, the premotor potential in these patients, presented as brief negativity starting 100–200 ms before the onset of movement, resembled the late BP, or BP2, potential and is similar to MRCPs shown in voluntary movements made in response to a triggering stimulus (64). Therefore, it is proposed that tics might be an internally driven movement, performed as a result of internal stimuli, and thus by-passing area 6, the brain area involved in the generation of early BP (65).

### Functional Movement Disorders

Functional movement disorders are under the umbrella term of functional neurologic symptom disorder (FNDs), which is defined by the Diagnostic and Statistical Manual of Mental Disorders, fifth edition (DSM-5) as neurological symptoms that are unexplained by other traditional neurological or medical conditions (55). FND is a preferential term to a wide variety of terms that have previously been used, namely, psychogenic, hysterical, conversion, and somatization, as it reflects a deeper understanding of the pathophysiologic aspect, shifting away from focusing highly on psychological precipitants as seen in Freudian conversion theories (66). Patients with FNDs can manifest a variety of movement types that often mimic neurological movement disorders such as tremor, myoclonus, dystonia, tics, and parkinsonism, but exhibit physiological characteristics that imply voluntary control, including variable or inconsistency (fluctuate in pattern, degree, and distribution); incongruous (difficult to explain by a known type of involuntary movement); distraction (decreases or disappears when the attention of the patient is drawn away from the movement); entrainment (movement characteristics cannot be maintained during contralateral competing movements); suggestibility (movement might start or stop or move to another body part as the suggestion of the examiner); or the presence of a cortical potential characteristic of self-paced voluntary action. Yet a hallmark of FNDs is that they are experienced, and subjectively reported by the patient as, completely involuntary (67, 68). While anxiety, depression, and personality disorders might be frequent co-occurrences, these features are not identifiable in every patient (69, 70). Moreover, these disorders are also seen in other neurological patients, so there is not a mandatory correlation and thus, psychological stressors and psychiatric comorbidity were removed from the diagnostic criteria in the DSM-5 (55, 71).

Advances in imaging techniques have provided new insights into differences in brain activity, functional connectivity, and brain structures in FNDs that are not detected on a structural MRI. For example, a voxel-based morphometry analysis showed abnormalities in the limbic area structure (72). fMRI and PET demonstrate abnormalities in regional brain activation and functional connectivity that mediate emotional regulation and awareness (ACC, vmPFC, insula, amygdala, and vermis) (73), executive and cognitive control including motor inhibition (ACC, dlPFC, and inferior frontal gyrus) (74, 75), motor planning (SMA), and perceptual awareness (PCC/TPJ) (31, 32).

Edwards et al. have proposed three key concepts in the neurobiology of FNDs which are attention, beliefs, and agency (76). First, FNDs look like a movement that has been consciously produced by the patient because attention is required to manifest the movements (77), and movements worsen when attention is drawn toward them, typically during physical examination (78). Electrophysiology in patients with FNDs has found evidence for a BP similar to that can be seen with voluntary movements, though, the presence of BP does not always indicate that a movement is voluntary (65). Indeed, prefrontal activation which represents movement-related attention has also been demonstrated in FNDs (79). Another concept is that of symptom-related beliefs and expectations which play an important role in altering sensory experiences. Patients with FNDs usually overestimated their symptoms according to Parees et al. who found that patients with functional tremor significantly overrated tremor duration in self-reported diaries compared with actigraphy (80). They have interpreted this finding within a Bayesian framework as an abnormally strong prior expectation, proposed to reside within SMA, over relevant bottom-up sensory data, and thus without appropriated and mismatch top-down control, this might result in the inappropriate selection of action in FNDs (65). The third key concept relates to SoA, which arises from the appropriate match of volition and movement feedback, likely centered on the parietal area. Mismatch in this process is misinterpreted by patients as without agency, and thus not self-generated which is a hallmark of FNDs.

### Stereotypies

Stereotypies lack a clearly defined terminology, yet continue to be debatable. One current workable definition by Edwards et al. defines them as a non-goal-directed movement pattern or vocalization that is repeated continuously for some time in the same form and on multiple occasions, which is typically distractible (81). Further confusion is generated by the attempt to classify stereotypies as voluntary or involuntary. One commonly quoted definition by Jankovic names them as being involuntary movements (3), however, this raises further questions, as stereotypy can be suppressed by distraction meaning that stereotypies require attention to manifest. Furthermore, it is probably impossible to obtain accurate reports from affected individuals as they are often very young children or persons with impaired mentation or learning difficulties.

Stereotypies can be classified according to their phenomenology as simple (e.g., leg shaking, foot tapping, hair twirling, nail biting, teeth grinding, body rocking, etc.) or more complex (e.g., hand waving, repeatedly opening and closing hands, hand posturing, head nodding, headbanging, repeatedly sitting down and getting up from a chair, finger wagging, pacing, orofacial movements, self-biting, and other self-injurious behaviors) (81, 82). Occasionally, vocal or phonic stereotypies (e.g., moaning, humming, grunting, or repeating words and phrases) can also be presented (83). From an etiologic point of view, stereotypies are divided into 2 categories, either primary or secondary, depending on the presence or absence of additional neurological or psychiatric disorders (83). Primary stereotypies appear to be purely physiological, i.e., they occur in normally developing children, however, subtle developmental issues have been noted (84, 85). It has been estimated that the prevalence of stereotypies in normal children is around 20%, with a typical age of onset by 3 years of age (86). The natural course is variable, with some declining after age 4 years, some persisting for multiple years (remained stable, improved, or became worse), and some even presenting into adulthood when stressed (86, 87). Common physiological stereotypies in adults are leg shaking, i.e., leg stereotypy disorder (88), playing with pens or hair, face touching, nail biting, hand or foot tapping, and body rocking (82). Secondary stereotypies, on the other hand, most often occur in children with autistic spectrum disorders, intellectual disability, or other neurological problems whereas stereotypies in adults are associated with various conditions, namely, drug taking, cerebrovascular diseases, namely, the frontal lobe, neurodegenerative diseases, infection, autoimmune encephalitis, and psychiatric conditions (89).

The underlying pathophysiology of stereotypies remains unknown. Psychological hypotheses had been proposed, including disorders of arousal and motor control, learned behavior, or as a component of underlying psychiatric disorders (90, 91). However, there is objective evidence supporting the involvement of underlying neurobiological abnormalities, ranging from structural brain imaging to neurophysiological studies. Reductions in frontal white matter and caudate nucleus were reported in healthy children with complex stereotypies (92). Other studies reported focal basal ganglia lesions resulting in stereotypies (93, 94). Looking into cerebral activity, unlike voluntary movements and FNDs, stereotypies that were not preceded by MRCPs suggest their physiologically distinction from that of voluntary movements (95). These results imply that stereotypies arise without normal control from premotor areas and indicate dysfunction within prefrontocorticobasal ganglia circuits (see Figure 2). Instead, this motor activity probably originates from the basal ganglia, related to the striatal dopaminergic system in particular, given that stereotypies were induced in rodents administered with dopamine D1 receptor agonists (96–98), and improved with D1 receptor antagonists (96, 99). Similarly, they have been found as a side effect of dopaminergic drugs, such as amphetamine, cocaine, and levodopa in clinical observation (97).

### Perseveration

Perseveration refers to the inappropriate continuation or repetition of response or activity (e.g., behavior, word, thought, strategy, or emotion). This term was first used by Neisser in 1895 and, since then, many clinical researchers have attempted to describe perseveration in several different forms and in association with a variety of neurological disorders and many underlying hypothetical mechanisms (100). However, there continues to be a lack of agreement in the literature as to how it should be classified. In this article, the authors have reviewed descriptions and classifications according to Liepmann (101) as this definition has been wildly used when describing perseveration in the form of movement disorders. He proposed three types of perseveration: (1) intentional perseveration, which is the repetition of previous response to a subsequent stimulus; (2) tonic perseveration, which is the inability to discontinue an action, e.g., unable to release another's hand after shaking it; and (3) clonic perseveration, which will be discussed in this section, a continuous repetition of an action that can be induced by passively moving a body part or by an external cue and its persistence occurs even after cessation of the external cue, e.g., continue to draw multiple loops when asked to draw once (101). The ability to induce the movement is a key to identifying and differentiating this phenomenology from stereotypies. Clonic perseveration is known to be a sequela of brain damage and typically occurs in a setting with a reduced level of consciousness, aspontaneity, usually with mutism and frontal releasing sign (102, 103). In those who relatively preserved language and speech, verbal perseveration, characterized by inappropriate repetition of phrases and words, was noted (103).

In considering neuroanatomical correlation, perseveration is commonly viewed as a cardinal feature of prefrontal pathology (102, 104, 105), however, it has been documented in thalamic (103, 106) or subthalamic infarction (107), and the fiber tracts connecting the limbic system to the frontal cortex (103). Therefore, it is suggested that perseveration occurs as a result of disconnection of the prefrontal corticobasal gangliathalamocortical loops that are important for the termination of motor plans (108–110).

### Behaviors That Mimic Semivoluntary Movements

The clinical distinction between movement and behavior is usually obvious. Movement is defined by the act of moving, whereas behavior defines how one acts and especially one's actions toward others (111). However, from a phenomenological viewpoint, there are some borderline forms in which repetitive unintentional behavior can mimic repetitive semivoluntary movement. Included are (1) compulsions, (2) utilization behavior (UB), and (3) motor mannerism.

Compulsions are characterized by repetitive and excessive behaviors that one is compelled to perform in response to intrusive and uncontrollable thoughts, which are labeled obsessions. These obsessive thoughts and compulsive behaviors are key components of OCD. More recently, DSM-5 recognizes four major types of obsessions: (i) obsessions related to fear of contamination associated with washing rituals; (ii) obsessions linked to harming focused checking rituals; (iii) obsessions of symmetry or order associated with counting rituals; and (iv) obsessive ideas linked to concerns about sex, religion, aggression, and other matters, associated with checking behaviors and/or purification rituals (55). The phenomenology of compulsions is complex and varied performing to reassure or counteract obsessions, for example, excessive washing or cleaning to remove germs, praying to counter sacrilegious thoughts, checking for assurance that doors are locked or people are OK, putting things in order (arranging), repeating other behaviors to get rid of a thought, etc. (112). In clinical practice, however, the boundary between compulsions and tics particularly complex motor tics, such as repeating actions until it feels right, is not easily determined. In addition, the frequent comorbidity between OCD and tics or Tourette Syndrome have been reported in which 20 to 60% of Tourette Syndrome sufferers display OCD symptoms and studies of patients with OCD have found tics in more than 50% of cases and Tourette Syndrome in 15% of cases (113, 114). For the clinician, certain distinctions, namely the content of obsessions, the nature of compulsions, the functional relationship between obsessions and compulsions, and the response to treatment are potentially useful discriminators for decision making (115).

According to the recent neurobiological model, OCD is characterized by the aberrant activity of the CSTC pathway in terms of overall hyperactivity of the corticostriatal loop (direct loop), in consequence, the indirect loop is no longer able to regulate or inhibit impulsive behavior and actions that are no more relevant or adequate to the situation. This results in a cortical hyperactivation leading to the typical symptoms of OCD, e.g., impulsivity and impaired action inhibition (116, 117). Concerning brain activity, previous studies that recorded event-related potential component of BP in patients with OCD found that they presented with a greater BP1 slope gradient and amplitude over bilateral frontoparietal areas corresponding to the motor cortex, which support the hypothesis that stronger motor response preparation for external stimuli might characterize OCD (118, 119).

Utilization behavior refers to a disorder in which patients automatically use or manipulate objects presented in the field of vision in an “object-appropriate” manner which is inappropriate for a given context (120). For example, when shown a toothbrush, a patient is likely to pick it up and begin to brush his teeth correctly, but in a context in which brushing teeth would not normally be done, e.g., in an appointment with a doctor. Moreover, patients usually claim that they intended to do so, as they do not detect a mismatch between actions and intentions. UB has been being recognized as an abnormal behavior following dysfunction of frontal areas or system, such that the top-down control of the frontal lobe can no longer inhibit the dependency of the parietal lobe upon sensory or environmental input (120, 121). Therefore, UB is another type of environmental dependency, as a result of an imbalance between frontal and parietal lobes.

Motor mannerisms are another unusual repetitive, distinctive behavior in an exaggerated and bizarre fashion, for example, eccentric postures, gestures, facial expressions, peculiar speech, unusual appearance in clothing, or makeup, etc. (122). They are considered goal-directed actions carried out by individuals in an attempt to call attention to themselves (122). Although mannerisms do not usually interfere with life, or cause self-injury, they can lead to withdrawal from, and rejection by, society. Mannerisms occur frequently in schizophrenia, however, they can be found in normal subjects, especially during puberty and adolescence, abnormal personalities, and neurological disorders (123).

Like semivoluntary movements mentioned earlier, mannerisms are under voluntary control but are displayed unconsciously, but the main difference is that mannerisms appear overdrawn and are considered to serve a distinct purpose, which is primarily communicative (124).

The pathophysiological mechanisms underlying manneristic behaviors are far from being understood. Some neurophysiological results suggest abnormal behavior-physiology interaction, secondary to pathological alteration of the brain metabolism, and disturbed perceptivity of social signals may be involved (125).

## Conclusion

The study of voluntary control is an integral part of the study of human movement. However, there is very limited information and tests available that allow distinctions between voluntary and involuntary movements to be made with confidence. A spectrum of semivoluntary movements exist in which patients may experience a disordered sense of will or agency, and thus a movement is experienced as unexpected and involuntary, for an otherwise voluntary-appearing movement.

The, as yet unclear, pathophysiological mechanisms underlying these conditions require further investigations, however, several functionally distinct areas within the frontal cortex before the primary motor area have been identified. These motor areas are differentially involved in movements made under different conditions. Table 1 summarizes different types of semivoluntary movements and associated behavior manifestation and several critical components, namely, semiology, common presentations, and neuroanatomical and neurophysiology pathologies to better distinguish between each condition and facilitate communication among clinicians and with patients.

**Table 1:** Different types of semivoluntary movements with associated behavior manifestation.

	**Semiology**	**Common features**	**Neuroanatomy**	**Neurophysiology**	**Sense of agency**	**Driven**	**Clues**	**Associated disorders**
Tics and tourette syndrome	Brief intermittent and repetitive movement. Varies; simple/ complex. Most frequently involving head and upper body.	Eye blinking, head jerking, shoulder shrugging grunting, throat clearing Echolalia Coprolalia Palilalia	Multiple brain areas and complex pathways.	No BP1	Normal	+/– Internal (premonitory urge)	Completely/ partially suppressible, persists during sleep	Attention deficit hyperactivity disorder, obsessive compulsive disorder, anxiety, depression
Functional movement disorders	Variety of movement types mimic neurological movement disorders but exhibit physiological characteristics that imply voluntary control.	Functional tremor, functional dystonia, functional myoclonus, functional parkinsonism, etc.	Right TPJ hypoactivation	Normal BP	Impaired	Distinct purpose, primarily communicative	Positive signs: - Inconsistency - Incongruous - Distractible - Entrainment - Suggestibility	Anxiety, depression, personality disorders
Stereotypies	Repetitive, non-goal direct movement that occur in a specific pattern and are distractible.	Leg shaking, foot tapping, hair twirling, nail biting moaning, humming, repeating words and phrases	Prefrontoco rticobasal ganglia circuits	No BP	Impaired	–	Distractible, Frontal release signs	Autistic spectrum disorder, mental retardation, other neurological/ psychiatric problem
Perseveration	Continuous repetition of an action induced by an external cue but Persist long after the cue stops.	Inappropriate repetition of words and phrases	Prefrontal pathology	Unknown	Unknown	Passively moving a body part or by an external cue.	Frontal release signs	Brain damage
Compulsions	Repetitive, excessive behaviors that compelled to perform in response to obsession.	Excessive washing or cleaning, praying, checking for assurance, putting things in order (arranging)	Hyperactive corticostriato thalamocortical loops	Greater BP1	Normal	Obsession- intrusive and uncontrollable thoughts		Obsessive compulsive disorder, Tics/ Tourette Syndrome
Utilization Bahavior	Automatically appropriate manipulate objects in view, but in an inappropriate context.	use the objects in appropriate way, but in an inappropriate situation	Prefrontal pathology	Unknown	Normal	Surrounding objects	Frontal release signs	Frontotemporal dementia, major depression, attention deficit hyperactivity disorder
Motor mannerisms	Repetitive, goal-directed, distinctive behavior in an exaggerate and bizarre fashion.	Eccentric postures, gestures, facial expressions, unusual appearance in clothing, or make-up	Unknown	Unknown	Normal	Social interaction	Biologically inappropriate and maladaptive, induce negative social responses	Schizophrenia Catatonia

*BP, Bereitschaftspotential; TPJ, temporoparietal junction; SMA, supplementary motor area*.

Semivoluntary movement is much more common than currently thought; given that most patients do not seek medical attention because of lack of resulting disability or misinterpretation as psychogenic in origin. Arguably, their recognition is clinically relevant since they are usually associated with severe conditions such as neurodevelopmental and neurodegenerative disorders.

Furthermore, limited improvement for these conditions has been reported with existing pharmacological therapies, and more effective treatments are needed. We hope that this review lays the foundation for stimulating discussion that will lead to a more comprehensive study, as a prerequisite for the discovery of improved pharmacologic interventions is understanding the underlying pathophysiology, epidemiology, including biological mechanisms.

## Author Contributions

SV and RB contributed to the conception and design of the study. SV organized the database and wrote the first draft of the manuscript. RB wrote sections of the manuscript. Both authors contributed to manuscript revision, read, and approved the submitted version of the manuscript.

## Funding

RB is supported by Senior Research Scholar Grant (RTA6280016) of the Thailand Science Research and Innovation (TSRI), the International Research Network Grant of the Thailand Research Fund (IRN59W0005), the Chulalongkorn Academic Advancement Fund into its Second Century Project of Chulalongkorn University, and the Centre of Excellence Grant of Chulalongkorn University (GCE 6100930004-1), Bangkok, Thailand.

## Conflict of Interest

The authors declare that the research was conducted in the absence of any commercial or financial relationships that could be construed as a potential conflict of interest.

## Publisher's Note

All claims expressed in this article are solely those of the authors and do not necessarily represent those of their affiliated organizations, or those of the publisher, the editors and the reviewers. Any product that may be evaluated in this article, or claim that may be made by its manufacturer, is not guaranteed or endorsed by the publisher.
